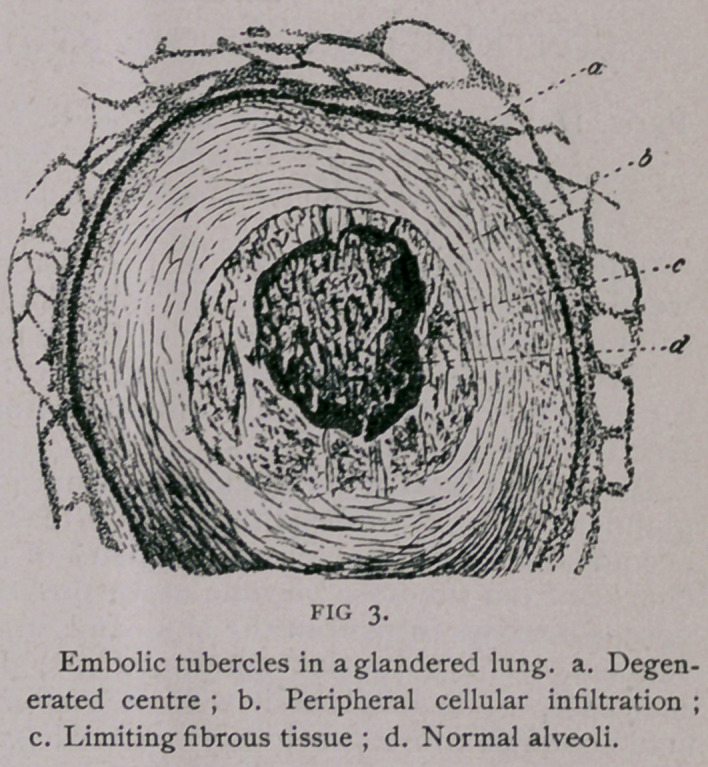# Calcified Fibrous Nodes of the Liver and Lungs of the Horse*Monatshefte fur praktische Thierheilkunde.

**Published:** 1892-03

**Authors:** S. J. J. Harger

**Affiliations:** Professor of Anatomy and Zootechnics in the Veterinary Department of the University of Pennsylvania


### CALCIFIED FIBROUS NODES OF THE LIVER AND THE LUNGS OF THE HORSE, AND THEIR DIFFERENTIATION FROM THE TUBERCLES OF GLANDERS.*

By S. J. J. Harger, V. M. D.

*Professor of Anatomy and Zootechnics in the Veterinary Department of the University of Pennsylvania.*

The presence of calcified or dense fibrous nodes in the liver of the horse frequently leaves the veterinarian in doubt as to whether they are due to glanders or to some other process. Glanders tubercles of the liver are always embolic (secondary), the bacilli being carried there by the blood vessels from some other organ which is the primary seat of the disease. This is usually a broncho-pneumonia caused by glanders, the microbe entering the pulmonary veins through a rupture, passing into the left side of the heart, and thence into the aorta and the hepatic artery; bacilli from other organs of the respiratory apparatus or the skin may enter the veins, and passing through the right side of the heart and the pulmonary arteries, some are arrested in the lungs, while others, from their small size, pass through the capillaries into the left ventricle, and finally reach the liver. If the liver alone contains nodules, without lesions in other organs, glanders must be eliminated.

The microscopic appearance of tubercles, glanderous or otherwise, of the liver, are in many respects similar. The former are whitish or grayish-white, circumscribed, creaking under the knife, compact and dry on section, not truly calcified, and contain a cheesy mass in their centre easily compressible under the finger nail. This mass, treated with acetic acid, does not give off bubbles of gas, rising under the microscope, as in the case of calcified tubercles; at most, only isolated ones can be produced by the process of teasing. When fresh, they appear greasy on section; situated between the hepatic lobules, they vary in size from a large pinhead to a pea. On a microscopic section the embolic glanders tubercles have the following characteristics: A peripheral circle of closely-packed cells, then a lighter granular layer from a commencing necrosis, and a centre showing complete necrosis with the well-known small granules. The last, stained with hæmatoxylon,

* Monatshefte fur praktische Thierheilkunde.

appears as a dark spot in strong contrast with the surrounding slightly granular, flaky, and lightly stained zone. In such conditions, other portions of the liver usually present many microscopic cellular infiltrations, small inflammatory nodules, caused by wandering bacilli. The fibrous zone surrounding old tubercles and forming a sort of capsule may, as in benign nodes, be seen in the more recent crops ; yet, in glanders, the regional infection by infiltration of young cells, small inflammatory foci can be distinguished around the connective tissue boundary of the older new formations. It is different in non-glanders calcified fibrous tubercles so frequently observed in the liver, which, disseminated under the serous covering, and the substance of the organ, in large numbers, sometimes singly, sometimes in groups of ten to twenty, of the size of a pinhead to a millet seed, and of a yellow or a yellowish-brown color, are in strong contrast with the dark color of the liver. These can be recognized as nodular aggregations, sandy and granular to the touch, hard to cut, and often surrounded by a grayish-white zone. The origin of these nodes is not yet known. Dickerhoff attributed them to vegetable parasites which reach the liver through the blood vessels, but does not explain their nature nor the source of the primary invasion of the blood. As they appear only as dry, necrotic nodules containing no foreign bodies, no fungi, it is difficult to assign their cause. They are, microscopically, dissimilar, aggregated, sometimes wedge-shaped nodes, sharply delimitated from the liver substance, and consisting of a thrombus-like mass of shrivelled-up, round, spindle-shaped and hepatic cells. The uniform aggregations of necrotic cells form a very coherent, homogeneous mass, so that in a microscopic section the nodule falls out. The liver tissue surrounding them, sometimes normal, sometimes shows newly-formed interstitial connective tissue, as in distomatose, which forms around the nodule a sort of capsule of spindle cells and fibres prolonged between the hepatic lobules and causing their atrophy. At times the centre of this necrotic mass is calcified, and it, as well as the bordering lobules, infiltrated with brown pigment. The cellular infiltration, which shows the spreading of the diseased process in the glanders tubercles, is absent here, and the necrotic parts are thrombi, which are soon mixed with leucocytes, the whole forming a uniform solid mass.

These nodes could be regarded as embolic infarcts of the smallest calibre, resulting from a thrombus of an abdominal artery or of the portal vein and its afferents. Where only the obviously old lesions are found, not the transitional changes of a fresh infarct, nor those of more recent formation, and as benign emboli in the liver can scarcely produce necrosis on account of the extensive collateral circulation, it is possible that they are old cicatrised necrotic nodes which could result from an omphalophlebitis in the foal.

Not every umbilical phlebitis leads to abscesses and pyæmia. It is fortunate that, according to the nature of the infecting material, which may reach the liver through the umbilical vein, and the varying degree of malignity of these emboli, in most cases, only small abscesses and necrotic nodes result, causing no essential disease, but eventually cicatrising in the form of nodular foci. The simultaneous existence of embolic nodes in the lungs and in the liver of the horse, shows that the passage of the embolic material from the umbilical vein into the lungs is possible, even if the ductus, Aurantii, be absent, when these particles are small enough to pass through the hepatic capillaries into the hepatic veins. The corpuscular elements here constituting the emboli are, of course, very small in omphalophlebitis, for the inflammation is suppurative, and the contained thrombus undergoes suppurative softening ; the portal vein, receiving the umbilical vein, carries the infectious material into the capillaries of the liver. As the nature as well as the virulence of this material vary, the emboli will have different effects—sometimes abscesses, sometimes minute dry necrotic masses, or fatty degeneration in small points, repaired in post-fœtal life during the growth of the liver by the formation of a fibrous capsule. In the same category with the preceding calcified nodes of the liver, which could, perhaps, be designated nodosis embolica, chalicosis fibrosa nodosa, the rare cases of total calcareous infiltration of the liver probably belongs. One instance of this kind is reported by Csokor, and a second one is preserved in the museum of the Munich veterinary school. The latter, presented by district veterinarian, H. Hess (Pasing) (April 17, 1887), is the liver weighing 16 kgms.. obtained from a horse ; the abdominal aorta to the root of the great mesenteric arteries was the seat of an aneurism, as large as an arm, continuous into the abdominal arteries ; the blood vessel walls were partially calcified. The liver, hard and enlarged, was roughened on its superficial surface like a grater, and could be cut only with the greatest difficulty. The whole organ was densely infiltrated with calcareous, sand-like, mostly round, sometimes oval nodules appearing like white points, surrounded by a delicate connective tissue layer, in the brown liver substance. The calcareous infiltration was so extensive that a section of the organ cut out shrunk but little, and the small calcareous points appeared like the rough side of a piece of sand-paper.

Microscopically, the incrusted parts showed a structure quite similar to that of the before-mentioned disseminated calcareous fibrous nodes ; they appeared like numerous embolic thrombi of blood plates, fibrin, red and white blood corpuscles ; the liver tissue between the thrombosed blood vessels was absorbed and the emboli themselves partially calcified. The thrombosis of the aorta proved the origin of the emboli. The liver described by Csokor weighed 20 kgms., was much enlarged, and the edges irregular ; the external surface bulging, and of a dark-brown color, was covered by glistening white nodes the size of a poppy—to that of a hemp-seed. The consistency of the liver was like that of stone, and could not be cut with the knife. A section with a saw had a speckled appearance, like limestone, in which numerous glistening, white, hard nodules were visible in the atrophied tissue of the liver; some had a mulberry-like form surrounded by a capsule. The thickness of the organ was 20 cm., the width 40 cm., and the tranverse diameter 75 cm. The isolated nodules, says Csokor, were situated between the hepatic lobules which were atrophied, the first effect of the new formation. As to their probable ætiology, no remark is here made.

It can not, at present, be shown that the calcareous nodes of the liver, as well as the more extensive incrustation of the same could be produced by destructive echinococci or cœnuri. In general, the calcification of echinococci takes place only after they have reached considerable size, and effects the granulation layer of the scolex so that the form of the vesicle is generally preserved, and can be eneucleated in the form of a hollow shell. The calcification here is so considerable that it appears like a stony-hard concretions the size of a hazelnut, of a pale-yellow or white color, with a hollow cavity containing a serous fluid or a turbid pulp. Such an instance is on record in the catalogue of the pathological collection of the Munich school, and another mentioned by Leuckardt. According to the latter observer, the wall of the echinococcus in man is calcified ; according to Klebs (Allgemeine Pathologie, 1888, § 243), the parasite itself is not calcified, but the capsule gives off trabeculæ of fibrous tissue ; other authors (B. Prongeanski) found these trabeculæ calcified. Osterstag, who recently contributed valuable literature on the echinococcus multilocularis in heifers and pigs, as well as Guillebeau, mention calcification of the central half of the connective tissue compartments and the caseation of the echinococcus mass.

I was long in doubt as to whether the above-mentioned calcified fibrous nodules and the total calcification of the liver were not due to the presence of cestodes, because the existence of the necrotic mass, with round cells and granules, could precisely be produced in the same way, only the complete absence of their calcified body. the scholices and the vesicles were contradictory to this view. Likewise, in echinococcus multilocularis, from their small size and retrograde metamorphosis, such results could follow, but here the multiplicity of the smallest vesicles, and the persistence of the characteristic lamellar structure are always distinguishable features.

If the undecided question, whether the echinococcus multilocularis belongs to a particular species of tænia, distingushed from the ordinary echinococcus, finds solution, and new research as to the details of their immigration into the horse made, the presence of cacified nodes in the liver of this animal may reveal some new facts.

Of the various cases of nodular new formations in the lungs of the horse, the most frequent is that of numerous calcified fibrous nodes, disseminated throughout the organ, and known under the general name chalicosis nodosa pulmonalis. One might, perhaps, as well name it nodosis pulmonalis fibrosa petrificons, fibro-tubercular lung-induration, and nodular cirrhosis. As the term cirrhosis is incorrect, the word tuberculosis utterly inapplicable, and Latin and Greek terms undesirable, a diagnostic nomenclature dependent upon the anatomical alterations remains to be found.

The cause of these nodes can not, in every case, be recognized, and as the different causes give rise to the same alteration, calcified tubercles, similar in microscopic appearance, their ætiology also is variable. One time it is embolic chalicosis (Csokor), another time koniosis (Martin), a third time cestoidean tuberculosis, and a fourth retained secretion from a bronchitis (Hahn), (chalicosis embolica, coniotica, verminosa simplex).

The nodular formations can not, with a proper knowledge, be confounded with glanders tubercles. The anatomical diagnosis of glanders, when the entire cadaver, skin, and respiratory organs can be carefully examined, and especially when microscopic and bacteriological examination is possible, can be easily made. From numerous specimens of such lungs sent to the pathological laboratory of the Bavarian school with the question whether it is glanders, it would seem that the differential diagnosis between the two sadly needs revision.

In chalicosis nodosis pulmonalis, the *lung* contains numerous irregular disseminated nodular foci, mostly of the size of a pinhead to a hempseed, seldom that of a linseed or a pea. They may be visible throughout the whole lung, or only in the anterior or the posterior lobe, subplewral, or in the parenchyma; not all are attached to the bronchi, but most of them are situated in the walls of the alveoli. In other respects the lung is normal, collapsed and wrinkled, crepitating, soft, elastic, reddish-brown, light red or yellowish-red, with a transparent pleura. Still, the nodules may be situated in an inflammatory area. Their presence is more apparent to the touch than to the eye, to which they appear as white or grayish-white foci, easily visible over the red surface of the lung, and bulging out from the cut surface of the organ. The color of the latter is uniform and shows no microscopic alterations, such as are characteristic of the alterations of glanders and tuberculosis undergoing cheesy degeneration. The calcified centre of the nodule is sometimes visible to the naked eye as a white point. The nodules are very hard, like points of cartilage, can be easily eneucleated with the fingers, slip away from the edge of the knife, making their section difficult, and creak when cut ; they have no hyperæmic zone ; the bronchial glands are nearly always free from nodular or degenerative changes, one of the best diagnostic signs from glanders, although, in rare cases, I have seen the same calcified nodular foci in these glands and in the lungs simultaneously. Such a rare coincidence may complicate the differential diagnosis. One can only suppose that in embolic chalicosis the smallest emboli are disseminated in both places by the capillary anastamosis between the pulmonary and bronchial arteries, as well as being carried from the lungs to the lymphatic glands by the lymphatic vessels.

The bronchi, as far as they could be followed with the scissors, are in many instances free from pathological alterations. In some cases the nodules are seen in the walls of the terminal bronchioles, which are nothing else than bronchiols, which have become clogged by the inhalation of foreign matter, causing a nodular, circumscribed, fibrous thickening at this point. Dickerhoff and Martin, the latter having made it a special study, designate this condition peribronchitis nodosa.

Sometimes the easily eneucleated nodes are found in the form of a dry, crumbly, yellowish-brown, cheesy mass, the size of a hempseed or even a pea, surrounded by a fibrous capsule 1 mm. thick, which remains in situ. Again, they are often only sub-pleural, with only a few in the interstitial connective tissue of the lung, and confined to the sharp border of the lung where they can be lifted from their seat.

Nodes, the reult of a chalky deposit in the bronchial mucus or the exudation of an old bronchitis can be recognized, as C. Hahn remarks, on the superficial surface by a racemose arrangement (corresponding to that of the alveoli of the lungs). It is, perhaps, well to mention that in embolic infarction of this nature, the calcified foci are disseminated through the whole lung ; but it may happen, if the emboli be few, that only an isolated area supplied by a single branch of the pulmonary artery may be invaded. Recent lung emboli are easily recognized, as Csokor has mentioned, by a light-red or a dark-red zone surrounding them, which, in the old formations, is entirely absent.

The presence of thrombi in the right ventricle or auricle and the vena cava will also give evidence of the source of the emboli. This proof, however, is very uncertain, because the node may be the result of an embolus of years before from the umbilical or portal veins.

The differentiation of the lesions due to inhaling dust from the two preceding is not very easy.

Diseases caused by inhaling foreign particles (pneumoconiosis), by vegetable particles (phytoconiosis), or stone or lime dust (chalicosis lapidarum), are most common in the horse ; in man, those resulting from coal-dust (anthracosis pulmonum) are the most frequent, although the preceding are also seen.

Of the particles inhaled, some are arrested on the moist walls of the trachea and the bronchi, penetrating the mucous glands and goblet cells, some are carried backward by the ciliated epithelial cells, and others, passing into the bronchioles, are expectorated with the sputum (Klebs).

Small molecular particles, penetrating the cement between the cells lining the lung alveoli, enter the parenchyma. The mechanism of respiration, being pump-like in its action, allows the inhaled matter to enter the lymph spaces beginning between these cells (Arnold, Klebs). The smaller particles then enter the lymphatic circulation, some remaining in the lungs, others reaching the mediastinal lymphatic glands ; their further migration and removal is effected through the lymphatic circulation (itself dependent upon the movements of the lungs), in which the white blood corpuscles play an important part. These leucocytes (fresszellen), which, like scavengers, destroy and remove all kinds of small, foreign, unnecessary particles in the animal body, waste products, and micro-organisms, also seize these dust particles and carry them, in part, back into the alveoli, and in part through the lymph current into the lymphatic glands where they are filtered out. Some of the solid part of the lymph contents being deposited here, it follows the lymph sinuses of the medulla and the cortex, the true follicles remaining comparatively free from the foreign matter. Besides the mediastinal and bronchial glands which, from their great size, can be examined microscopically, the foreign bodies are also arrested by the small microscopic peribronchial and sub-pleural lymphatic glands.*

Recent experiments have shown that if foreign bodies, such as bacteria or mould fungi, enter the organism, leucocytes will collect around them, digest and destroy them, thus indicating, as is believed, that these migrating cells are, by some chemical property, called chemico-tactile, attracted to foreign bodies (A. Rosenberger, Pfeffer, Klebs). We have often observed that foreign bodies, irritating to the tissues but without toxic effects, such as needles, can become encysted without any visible reaction of the tissues. According to Buchner's researches, it has recently been demonstrated that bacterial protoplasm has a strong attractive force for leucocytes, a probable explanation of the abundant migration in inflammation. The larger microscopic sand and dust particles, which, from their weight, can not be destroyed by the leucocytes, obstruct the lymph vessels, or enter the lung tissue by an abraded surface, lodge there, and usually give rise to a circumscribed inflammation, terminating in a fibrous new formation; an extensive migration of white blood cells to the seat of the foreign body takes place; this cellular deposit leading to an inflammatory line of demarcation arrests this body and produces its encapsulation. From this results the formation of embryonic and granulation tissue, which is finally changed into a fibrous capsule. While in pneumo-coniosis fibrosa and in fibrous calcified nodes, new fibrous tissues develops concentrically around the foreign body; we also find as a result of dust particles, disseminated through the agency of the lymphatic vessels, streaks and bands of cicatritial tissue (lung cirrhosis, lymphangitis pulmon. fibrosa); an entire lobe may be thus involved, the epithe lium desquamating, and the alveolar walls uniting with each other (bronchio-pneumonia lobularis fibrosa). The diagnosis of this condition from that of a lung pigmented by coal-dust or appearing

* Sub-pleural lymphatic glands were first mentioned by Knauff, and also observed by J. Arnold in large numbers in the lung of man and of animals.

smoked is easy. In horses working in coal mines, anthracosis, with such discoloration, is frequent ; in dogs it is quite common in the form of small grayish-black points.

The origin of calcified fibrous nodes in the lungs is quite variable, and the macroscopy, being so uniform, does not give a clue as to the cause ; however, the whole group has characters distinctive from those of glanders tubercles. The former are characterized by a *uniform calcification of each node*, and hence the name chalicosis. Even admitting that glanders tubercles may calcify, a *general calcification of the nodes in an entire lung immediately excludes glanders.*

Professor Csokor, the celebrated pathologist, writes as follows : "I have never observed calcification of true glanders emboli, nor a deposit of lime salts into the connective tissue at their seat. When calcified tubercles were observed in a glandered lung, they appeared to me to be attributable to a previous cause."

Professor Hahn, who published an exellent article on glanders and its pathology in the yearly report (1869–70) of the Munich Veterinary School, is of the same opinion : "The unnourished centre (of the glanders tubercle) soon disintegrates into a dry, cheesy mass, and I never found it calcified ;" in over 300 cases of glanders observed by this author, calcified tubercles were never found.

Dickherhoff claims to have found in the centre of old glanders tubercles aggregated in masses as large as a hazlenut, a calcified area of the size of a hempseed or a pea, while their other parts were indurated. But further on he says : "Often the small tubercles of pulmonary glanders have a glassy, pearly aspect, in which condition they may remain for months, even years, without undergoing calcification."

That glanders tubercles do not calcify, which I believe is true, and that I have never in the broncho-pneumonia, embolism, and lymphangitis of glanders found calcification, is opposed to the old theory that the calcified tubercles shrink ; this is also mentioned in the latest text-books (Lehrbuch Pathologishen Anatomie von Birch Hirschfeld—Johne, 4. Auflage, 1889, and Friedberger—Fröhner, Lehrbuch der Speciellen Pathologie und Therapie, 1889). It is questionable whether these statements are only a repetition of the views of the older authors, or whether the observed cases were simply coincidences of glanders and nodular calcification of the catarrhal exudate of a pneumonia induced by glanders.

A more accurate differential diagnosis af these diseases can be made by microscopic sections cut with the freezing microtome.

Hardened in alcohol, stained, and imbeded in paraffine, nodes not calcified are not cut very easily with the better instruments, because they become too hard, brittle, and injure the edge of the blade ; previous calcification destroys the histological type. By means of the freezing microtome an excellent section of a non-calcified node can be made. Stained with hæmatoxylon, borax-carmine, and picro-carmine, the nodes are easily distinguished from the pulmonary tissue, and, under a low power of the microscope, appear as solid, circular, irregularly round islands, composed of fibrous tissue arranged concentrically. In chalicosis, this fibrous tissue in the form of a capsule, surrounds a various altered embolus. Now and then the thrombus shows the fibrin network, but usually it is organized, containing in its centre a larger or smaller number of round cells and fibroplastin traversed by capillaries.

In cases of calcification, the calcified point, after staining with hæmatoxylon, becomes dark, and is easily distinguished from the cellular, non-granular fibrous capsule ; with other reagents, the calcified parts are recognized by a marked bright and laminated aspect.

FIG I.

Calcified fibrous tubercle of the lung of a horse. a. Normal alveolus ; b. Fibrous capsule ; c. Slight calcification ; d. Centre completely calcified.

When a part of the nodes as well as the embolus are calcified, it is difficult to recognize the latter ; the central calcareous deposit destroys the identity of the embolus as an inhaled foreign body surrounded by a capsule. The calcareous deposit also invades the fibrous zone, whose bundles thus infiltrated become laminated, fissured and zigzag in comparison with the non-calcified tissue. The alterations are most obvious when, in transverse section, the calcified zone forms a ring around a non-calcified embolus. The absence of cartilage in and around the nodes, their situation along the bronchial tubes, assist in recognizing that they are obstructed blood vessels whose walls form a part of their capsule ; indurated and obliterated blood vessels are found in connection with the nodes. The latter process can be recognized as an embolic arteritis or vasculitis obliterans nodosa.

FIG 2.

Calcified fibrous node of the lung of the horse. a. Normal alveolus ; b. Fibrous capsule ; c. Zone of calcification ; d. Organized embolus ; e. Pulmonary vein.

In other cases of nodosis fibrosa petrificans, the microscopic alterations are similar, excepting the absence of the embolus ; in dust inhalation the embolus consists of fragments of stone, plants, and coal-dust, and, if the node is in the wall of the alveolus, must have lodged in a terminal bronchial or the interstitial connective tissue.

All these nodosities show *no peripheral cellular infiltration*, and produce no inflammatory changes other than the fibrous tissue proper to the new formation.

A glanders tubercle is microscopically distinguishable from all these nodular growths, be they primary or secondary, by emboli or through the lymphatics.

Csokor has most clearly and simply represented the structure of this tubercle, and I will reproduce it after him.

FIG 3.

Embolic tubercles in a glandered lung. a. Degenerated centre ; b. Peripheral cellular infiltration ; c. Limiting fibrous tissue ; d. Normal alveoli.

A transverse section of a glanders tubercle (Fig. 3) shows a fatty, granular central mass, whose inner portion is more finely granular stains as a dark spot, while its outer part, more homogeneous and less granular, stains less deeply. The whole mass lies internal to the alveoli, involving a group of them, so that their septa still remain visible, giving the section an areolor aspect.

The cheesy microscopic centre results from cell proliferation and their subsequent fatty degeneration as well as that of the alveolar epithelial cells; it represents the point of invasion of the virus; besides, the surrounding lung tissue is congested and infiltrated with cells Hence we find, around the degenerated centre, rows of alveoli filled with round small islands of round cells, ready to infiltrate the healthy tissue. While the infiltrated alveoli undergo a similar degeneration, a new inflammatory zone forms, which, in order to protect the healthy tissue, developes a fibrous capsule. Between the latter and the infiltrated layer there is an inflammatory exudation of cells, undergoing internally granular degeneration and externally fibrous transformation. Thus follow in succession degeneration, cellular infiltration, and limiting fibrous tissue ; the degeneration begins in the connective tissue and the tubercle gradually enlarges.

The preceding remarks, illustrated by micro-photographs, present the differences between nodosis fibrosis petrificans or benign fibrous nodes and glanders tubercles of the lung and the liver; they demonstrate the histological alterations under a low power of the microscope

## Figures and Tables

**FIG 1. f1:**
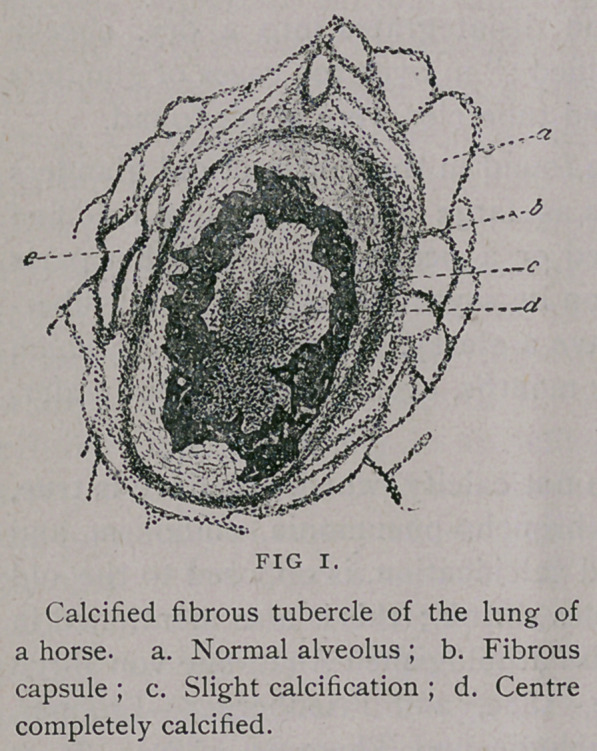


**FIG 2. f2:**
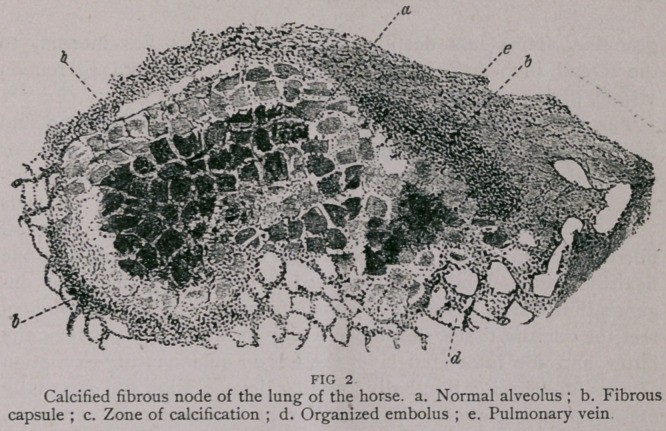


**FIG 3. f3:**